# Applications of PNA-Based Artificial Restriction DNA Cutters

**DOI:** 10.3390/molecules22101586

**Published:** 2017-09-21

**Authors:** Narumi Shigi, Jun Sumaoka, Makoto Komiyama

**Affiliations:** 1 World Premier International (WPI) Research Center for Materials Nanoarchitectonics (MANA), National Institute for Materials Science (NIMS), 1-1 Namiki, Tsukuba, Ibaraki 305-0044, Japan; SHIGI.Narumi@nims.go.jp; 2 Life Science Center of Tsukuba Advanced Research Alliance, University of Tsukuba, 1-1-1 Tennoudai, Tsukuba, Ibaraki 305-8577, Japan; sumaokajn@stf.teu.ac.jp; 3 Department of Applied Chemistry, School of Engineering, Tokyo University of Technology, 1404-1 Katakuramachi, Hachioji, Tokyo 192-0982, Japan

**Keywords:** PNA invasion, site-selective genome scission, site-selective DNA cutter, DNA/RNA hybrid

## Abstract

More than ten years ago, artificial restriction DNA cutters were developed by combining two pseudo-complementary peptide nucleic acid (pcPNA) strands with either Ce(IV)/EDTA or S1 nuclease. They have remarkably high site-specificity and can cut only one predetermined site in the human genome. In this article, recent progress of these man-made tools have been reviewed. By cutting the human genome site-selectively, desired fragments can be clipped from either the termini of chromosomes (telomeres) or from the middle of genome. These fragments should provide important information on the biological functions of complicated genome system. DNA/RNA hybrid duplexes, which are formed in living cells, are also site-selectively hydrolyzed by these cutters. In order to further facilitate the applications of the artificial DNA cutters, various chemical modifications have been attempted. One of the most important successes is preparation of PNA derivatives which can form double-duplex invasion complex even under high salt conditions. This is important for in vivo applications, since the inside of living cells is abundant of metal ions. Furthermore, site-selective DNA cutters which require only one PNA strand, in place of a pair of pcPNA strands, are developed. This progress has opened a way to new fields of PNA-based biochemistry and biotechnology.

## 1. Introduction

Site-selective scission of DNA is one of the key techniques in DNA manipulation and DNA nanotechnology [1]. In order to manipulate small DNAs (e.g., plasmids), naturally-occurring restriction enzymes have been widely and conveniently employed. In recent biology and biotechnology, however, still larger DNA (e.g., human genome) are the primary targets, and the site-specificity of restriction enzymes (recognition of 4–8 bp cognate sequence) is too low for site-selective scission. As a solution to this problem, nuclease domain of FokI restriction enzyme was conjugated with protein motifs which bind to predetermined DNA sequence (ZFN: Zinc finger nucleases [2] and TALEN: transcriptional activator-like effector nucleases [3]). RNA-guided site-selective scission of genome by a nuclease (CRISPR: clustered regularly interspaced short palindromic repeats-Cas9: CRISPR-associated nuclease 9 system) was also reported [4]. These protein-based DNA cutters are very useful for in vivo applications involving genome editing. For most in vitro applications, however, chemistry-based DNA-cutting tools have various advantages which cannot be fulfilled with protein-based tools (e.g., high stability for usage and storage, broadness of substrate structures, smaller sizes, versatile functionalization through chemical modification, and others).

**Figure 1:**
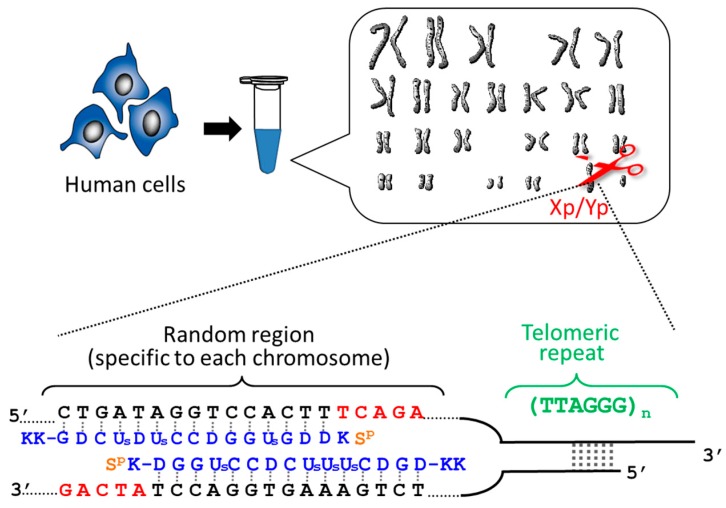
Clipping of individual telomere from each of the chromosomes in human cells. ARCUT is designed to cut the site where the telomeric repeats (TTAGGG)n terminate and the sequence is specific to each telomere (Xp/Yp here).

About 10 years ago, completely chemistry-based artificial restriction DNA cutters (ARCUT) were developed [5,6]. These cutters are composed of (i) two pcPNA strands (15-nucleobases each) which form double-duplex invasion complex at the target scission site; and (ii) Ce(IV)/EDTA complex which hydrolyzes only single-stranded DNA. The binding sites of two pcPNA strands are laterally shifted from each other by several bases (e.g., five nucleobases) so that single-stranded portions are formed at desired sites in both strands of DNA and are selectively hydrolyzed by Ce(IV)/EDTA (typical structure of ARCUT is presented in Figure 1). The DNA scission proceeds via the hydrolysis of targeted phosphodiester linkage, and thus the scission fragments can be straightforwardly ligated by ligase with other DNA fragments, rendering ARCUT super restriction enzymes which enable sequence-free and specificity-free cut-and-paste manipulation. These artificial DNA cutters recognize in total 20 bp sequence in DNA (one 10 bp sequence involving two pcPNAs + two 5 bp sequences involving one pcPNA). Accordingly, even human genome (3 × 10^9^ bp) can be selectively cut at only one site with this man-made cutter (4^20^ >> 3 × 10^9^) [7]. Furthermore, site-selective scission of a gene by ARCUT is successfully recognized by the repair system in human cells, and the gene is converted to another gene through homologous recombination [8]. For some applications, S1 nuclease can also be used in place of Ce(IV)/EDTA, although undesirable excessive digestion of the scission fragments could occur to some extent (vide infra) [5].

The basic concepts of molecular design of ARCUT and its excellent features for site-selective DNA scission have been already covered in several reviews [6,9,10,11]. Accordingly, this review will emphasize recent progresses, especially in the practical applications of these artificial DNA cutters.

## 2. Clipping of Desired Fragments from Genomes by ARCUT

One of the most promising applications of ARCUT is to cut genomes selectively at predetermined site(s) and obtain desired fragments from them. As is well known, genomes are chemically modified in complicated, but well controlled, manners to precisely regulate the biological functions (epigenetics). Typical modifications are 5-methylation and 5-hydroxymethylation of cytosine residues. Pyrimidine dimers are also formed by 2 + 2 photocycloaddition. Furthermore, various proteins (e.g., histones and other proteins) are binding to the genomic DNA. If we can clip desired fragments from genomes (with or without the proteins), highly-complicated functions of genomes can be analyzed in detail and our understanding of them is greatly deepened. Furthermore, the genomic fragments can be valuable frameworks for DNA nanoarchitectures [12,13,14], which have been growing with an enormous speed in this decade [1].

Several methods have been already proposed to prepare small fragments from genomes [15,16,17,18,19]. As an example, enzymatic digest of the genome was first treated with an exonuclease to provide a single-stranded portion at the end of DNA fragments, and target fragment was picked up by conjugating with a complementary DNA probe [15]. Alternatively, homopyrimidine/homopurine sequences in genomic fragments were targeted by triple-helix forming oligonucleotides for the separation [16,17]. However, there has been no established method in which desired fragments can be easily and efficiently isolated from genomes without critical limitations in their sequences and lengths. Note that we cannot use PCR methods to obtain these genomic fragments in their naturally-occurring forms, since epigenetic information (chemical modifications of genomes) is completely lost during the amplification processes by DNA polymerases.

### 2.1. Determination of the Length of Individual Telomere of Each Chromosome

Telomeres are located at the ends of chromosomes and serve as a “molecular clock” to determine cellular lifespan. When the lengths of telomere are shortened by cell division down to a critical size, the senescence or death of cells is triggered. Accordingly, telomeres play a pivotal role in genome stability, cancer, and aging. The simplest and the most fundamental question in telomere science is whether the telomeres of all the chromosomes in a cell have the same length or are different in length from each other. However, no concrete answer has been obtained, since all the previous methods simultaneous evaluate the lengths of all the telomeres in the cells. Thus, the length of an individual telomere at a single chromosome terminus can never be determined.

By using ARCUT, telomere of each chromosome in human genome (e.g., 11q, 12q, and Xp/Yp) was separately clipped out [20,21]. In order to obtain the telomere at Xp/Yp (short arm of the sex chromosomes), for example, the corresponding ARCUT was designed to selectively cut a site in Xp/Yp where the telomeric repeat (TTAGGG)n terminates (Figure 1). Although the telomeres of all the chromosomes of human beings have the same sequence ((TTAGGG)n), this site in the subtelomere region is different from chromosome to chromosome. When the whole genome of human beings was treated with this ARCUT, specific to the telomere of Xp/Yp, only the target site was cut so that the telomere of Xp/Yp was selectively separated. In order to obtain the telomeres of 11q and 12q selectively, the corresponding cutters were independently designed and prepared, and the whole genome was treated by them. It was found that the telomere length is notably different from chromosome to chromosome, even when they are obtained from the same cells and, thus, the number of cell division is exactly the same. This result could open a new insight to the role of telomere as a timekeeper of cells.

### 2.2. Preparation of Targeted Internal Fragments from Human Genome

**Figure 2:**
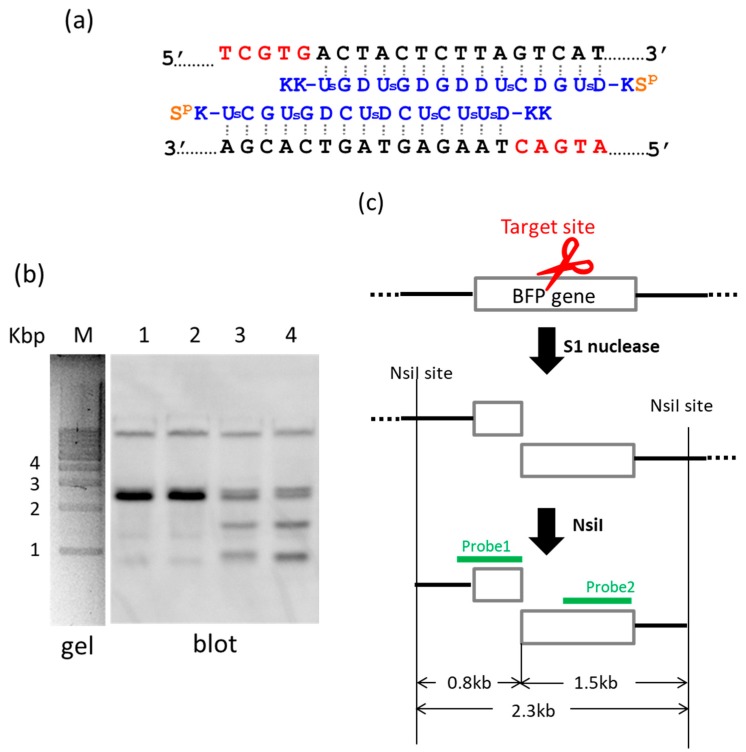
Clipping of internal fragments from human genome using the S1 nuclease–pcPNA combination. The genome was first treated with artificial DNA cutter (the combination of two pcPNA strands and S1 nuclease), and then digested by a restriction enzyme NsiI. The products were analyzed by Southern blotting. (**a**) The sequences of pcPNAs targeting the BFP gene which is stably integrated into the human genome; and (**b**) Southern blotting. Lane 1, treatment of the genome with S1 nuclease in the absence of the pcPNAs; lane 2, incubation of the genome with the pcPNAs in the absence of S1 nuclease; lane 3, the scission by using the S1 nuclease–pcPNA combination at 37 °C for 1 h; lane 4, the scission by the combination at 50 °C for 30 min; lane M, 1 kbp markers. As presented in (**c**), the dual scission by the artificial DNA cutter and NsiI should provide two fragments of 0.8 and 1.5 kbp, which were detected in the lower parts of the gels in lanes 3 and 4 in (**b**).

By using ARCUT, a targeted fragment in the human genome can be also extracted from the middle of genomes [22]. In Figure 2, S1 nuclease was used as the molecular scissors in place of Ce(IV)/EDTA. Desired internal fragments of the genome were clipped. First, the whole genomic DNA was extracted from human cells, and incubated with a pair of pcPNA strands. The single-stranded portions (the red nucleotides in Figure 2a) were formed by these two pcPNA strands at predetermined sites in both DNA strands, and were cut by S1 nuclease. The concentration of Zn(II), a cofactor of this enzyme, was kept low (5 mM), since this divalent metal ion stabilizes the DNA duplex and suppresses the pcPNA invasion. The scission products were purified by ethanol precipitation, and further digested by a restriction enzyme NsiI. As shown by Southern blotting in Figure 2b (lanes 3 and 4), two scission fragments were successfully clipped. The sizes of these clipped fragments (0.8 and 1.5 kbp) are exactly identical with the values expected from the dual scission by the artificial DNA cutter and NsiI (see Figure 2c). The band of the 2.3 kbp fragment is due to the dual scission of the genome by NsiI, without the scission by the ARCUT. Here, an ARCUT was combined with an appropriate restriction enzyme to facilitate the analysis of the products, but use of two different ARCUTs is also possible.

For this preparation method, easy availability and eminent biocompatibility of S1 nuclease are advantageous. The products of the present procedure can be slightly (e.g., several nucleotides) shorter than the targeted site-selective scission fragments, since even the double-stranded portions are to some extent digested by S1 nuclease from the ends. This is highly in contrast with the fact that Ce(IV)/EDTA (combined with pcPNA strands) never trims the termini, and thus the fragments of predetermined terminus structure can be precisely obtained [5,6]. In order to ligate the scission products with other fragments and prepare recombinant DNA of discrete sequence, the Ce(IV)/EDTA–pcPNA combinations are more appropriate. However, the S1 nuclease–pcPNA combinations should be very convenient tools especially for some biological and biochemical applications where the trimming, if any, is not a significant problem.

## 3. Site-Selective Scission of DNA/RNA Hybrids by ARCUT

**Figure 3:**
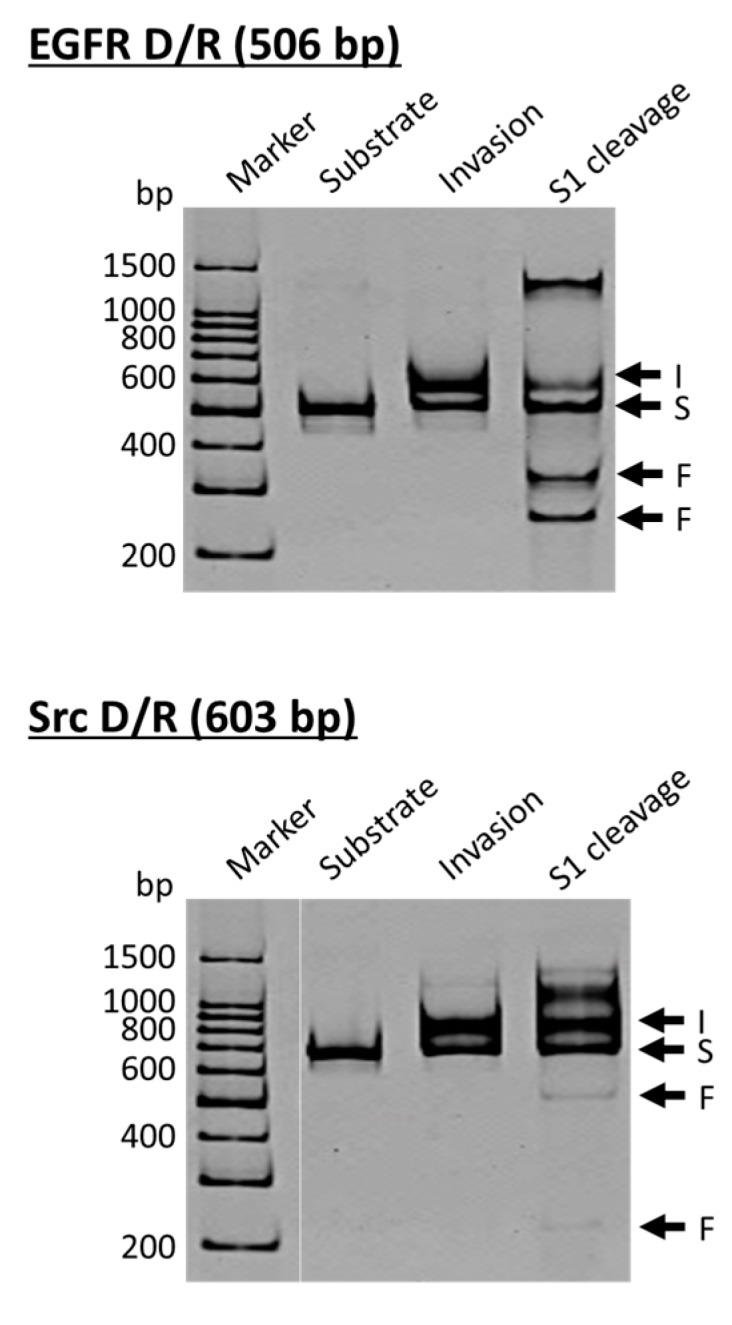
Site-selective scission of DNA/RNA hybrids by ARCUT. The DNA strand in the upper gel is a part of the epidermal growth factor receptor (EGFR; from human chromosome 7), whereas the DNA in the lower gel is from the proto-oncogene tyrosine-protein kinase (Src; from human chromosome 20). S, DNA/RNA hybrid; I, invasion complex; F, the scission fragments. Scission conditions: [substrate DNA/RNA duplex] = 10 nM, [pcPNA] = 50 nM, [S1 nuclease] = 9 units/μL, [NaCl] = 280 mM, and [ZnSO_4_] = 1 mM at pH 4.6 and 25 °C for 3 min.

ARCUT is a convenient and useful tool for site-selective scission of duplexes between a DNA strand and its complementary RNA strand, and also for the manipulation of DNA/RNA duplexes [23]. As molecular scissors, S1 nuclease is eminent and cut both of the DNA strand and the RNA strand in DNA/RNA hybrids selectively at the target site. In cells, DNA/RNA hybrids are formed in transcription, reverse transcription, and DNA replication, and have been regarded as important targets of small binding ligands for therapy [24,25]. These hybrids are also derived from pathogen, and recognized as a virulence factor to activate the host immune system [26,27]. In the upper side of Figure 3, the DNA strand is a part of the epidermal growth factor receptor (EGFR; from human chromosome 7), whereas the DNA in the lower side is from the proto-oncogene tyrosine-protein kinase (Src; from human chromosome 20). The duplexes of both DNA strands with the corresponding RNA were site-selectively hydrolyzed at the target site, providing two bands of the expected sizes (the bands designated as F in S1 cleavage lanes). As is the case for the scission of double-stranded DNA, the site of selective scission is a priori determined by the sequences of the pcPNA strands and almost freely chosen. According to detailed analysis, unique sticky ends are formed at the termini of the scission fragments. It is noteworthy that the scission fragments are sufficiently protected from trimming with S1 nuclease. Accordingly, they are directly ligated with other DNA/RNA hybrids by using T4 DNA ligase, providing desired recombinant DNA/RNA hybrids.

This fabrication method should be valuable, especially when the DNA/RNA duplexes cannot be directly prepared by using reverse transcriptase due to complicated tertiary structures of RNA template, its chemical modifications, or other factors. A variety of chemically-modified DNA/RNA hybrids can be also prepared by ligating the scission fragments with the hybrids formed from the corresponding modified oligonucleotides. Accordingly, the present method would pave the way to new DNA/RNA hybrid science. In place of S1 nuclease, Ce(IV)/EDTA was also effective for site-selective scission of DNA/RNA hybrids. However, the scission products were notably shortened at a longer reaction time. This result is highly in contrast with the digestion of the corresponding double-stranded DNA. There, Ce(IV)/EDTA precisely produces the targeted fragments, whereas S1 nuclease shows notable trimming (vide ante).

## 4. ARCUT for Gene Manipulation

Double-strand break of DNA by ARCUT is satisfactorily recognized by the repair systems in human cells and induces the corresponding homologous recombination [8]. Thus, the DNA manipulation by this tool can be useful to alter an intracellular gene in human cells to another gene and also to incorporate a desired extracellular gene into the cells.

### 4.1. Homologous Recombination in Human Cells

The efficiency of recombination is strongly dependent on the cell cycles, the introduction method of ARCUT into the cells, and many other factors. Thus, these experimental factors were optimized through chemical and biological approaches. Of two kinds of terminal structure formed by ARCUT, 3′-overhang termini are more appropriate than 5′-overhang termini (the terminus structure is governed by the direction of lateral shift of two pcPNA strands used). The recombination efficiency increases with increase in the homology length, as expected. Homologous recombination is further promoted by synchronizing the cell cycle to G2/M phase with nocodazole and also by repressing the non-homologous end-joining (NHEJ)-relevant proteins Ku70 and Ku80 by siRNA. It was indicated that appropriate combination of all these chemical and biological approaches should be effective to promote ARCUT-mediated homologous recombination in human cells. Direct scission by ARCUT at the target site of a predetermined gene resulted in the conversion of a target gene to another gene for genome editing in human cells [28,29].

### 4.2. Gene Insertion to a Target Site of Plasmid in Human Cells

ARCUT-mediated homologous recombination is further extended to the insertion of various genes into predetermined sites of plasmid in human cells [30]. The donors possessed only the sequence of either an internal ribosome entry site (IRES)-neomycin resistant (NeoR) gene or 2A-red fluorescent protein DsRed2 gene, but had no promoter (IRES and 2A were used to allow the expression of the corresponding proteins in human cells). The homology region in the donor covered about 190 bp of the upstream of the ARCUT scission site and 510–540 bp of its downstream. When the targeted homologous recombination occurred in the human cells, the IRES-NeoR gene cassette (1608 bp) or the 2A-DsRed2 gene cassette (754 bp) should be inserted to the plasmids. Successful expression of the inserted genes in the human cells was confirmed by either expected antibiotic resistance of the cells or their fluorescence emission. The efficiency of ARCUT-induced insertion of these long DNA donors was sufficiently high. The versatile utility of ARCUT for gene manipulation has been further indicated.

## 5. Second-Generation ARCUT for Still More Versatile Applications

Although pcPNA-based site-selective DNA cutters presented above (“first-generation” ARCUT) are highly promising for versatile applications in vivo and in vitro, there remain several factors that must be solved for still wider applications of these artificial tools. The most important points are (i) further extension of the scope of DNA sequences that can be site-selectively cut and (ii) successful site-selective scission under still more harsh reaction conditions. Both of these two factors are solved primarily in terms of promotion of PNA invasion which induces the formation of hot-spot of the scission (single-stranded portion) at target site.

### 5.1. Extension of the Scope of Sequence of Scission Site

Double-duplex invasion occurs without significant limitation in DNA sequences and, thus, the site for selective scission by ARCUT is relatively freely chosen. However, there are still several sequences that are difficult to be targeted by PNA invasion. The first one is highly guanine-rich (G-rich) sequences which take important biological roles and are strongly related to cancer [31,32,33]. For example, G-rich sequence in the promoter of c-myc oncogene controls the transcriptional activation, probably through the formation of G-quadruplex structures [34,35]. The invasion to these sequences is difficult, primarily because eminent pseudo-complementary bases for G and C are not available [34]. In G-rich sequences, the number of the pseudo-complementary bases D and Us for A and T in the corresponding pcPNA strands is intrinsically limited and, thus, the duplex formation between two pcPNA strands cannot be sufficiently suppressed.

**Figure 4:**
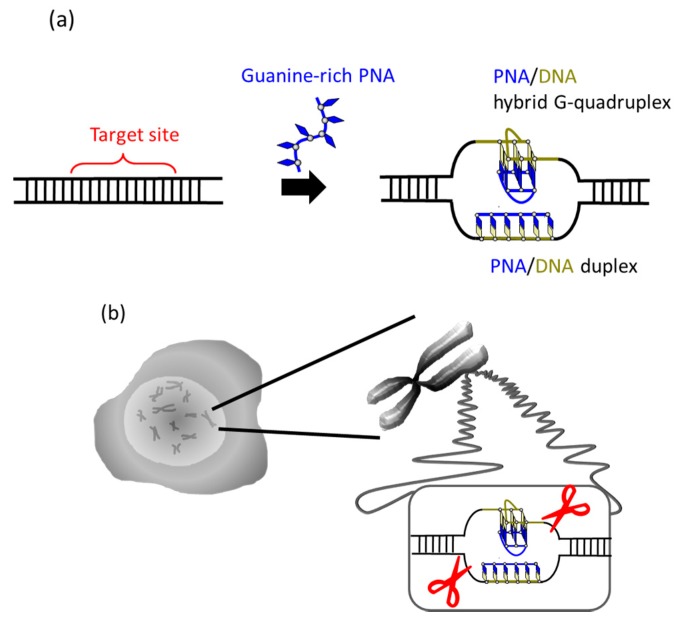
(**a**) Recognition of G-rich sequence by one strand of PNA and (**b**) its site-selective scission by Ce(IV)/EDTA. In (**a**), one PNA strand forms a G-quadruplex with the upper G-rich DNA strand, and another PNA strand forms Watson-Crick duplex with the lower C-rich DNA strand.

It was recently reported that a single PNA effectively binds to the G-rich region in double-stranded DNA through formation of a PNA/DNA hybrid G-quadruplex (Figure 4) [35]. A sequence in cancer gene (5′-AGGGTGGGGAGGGTGGGGA-3′/3′-TCCCACCCCTCCCACCCCT-5′) was targeted. Upon the addition of a G-rich PNA (GGGTGGGG), one strand of this short G-rich PNA formed a G-quadruplex with the G-rich sequence in the upper DNA strand (note that conventional T, not Us, was used). On the other hand, another strand of this PNA formed conventional Watson-Crick duplex with the counterpart C-rich sequence in the lower strand. Because of these two kinds of bindings, the targeted G-rich site was precisely recognized and activated for site-selective scission. When the system was treated with Ce(IV)/EDTA, the target site was selectively hydrolyzed. The result provides a new concept of targeting G-rich duplex DNA sequences through PNA/DNA hybrid G-quadruplex formation.

The next target is consecutive homopyrimidine/homopurine sequences which are also crucial for a range of cell functions (e.g., transcriptional regulation, chromatin organization, and DNA repair) [36,37]. Double-duplex invasion of pcPNA is practically inapplicable to these sites, since pcPNA strands involving consecutive purine sequences are very difficult to prepare by solid-phase synthesis (the coupling efficiency significantly decreases with increasing product length, probably due to high hydrophobicity of consecutive purine bases). Accordingly, a new site-selective DNA cutter was developed by combining triplex-forming PNA with Ce(IV)/EDTA [38]. Only one PNA conjugate involving conventional PNA (containing no pseudo-complementary bases) is required for the site-selective scission. The PNA involves two homopyrimidine sequences (connected by polyethylene glycol) and binds to the homopurine sequence in DNA through triplex formation. As the result, the homopyrimidine sequence in the homopyrimidine/homopurine site becomes single-stranded and is selectively hydrolyzed by Ce(IV)/EDTA. Increased versatility in the sequence for site-selective scission should widen the scope of applications in biology, biotechnology, medicine, and other fields.

### 5.2. Site-Selective DNA Scission under High Salt Conditions

High salt conditions are unfavorable for double-duplex invasion of pcPNA, since DNA duplex is stabilized under these conditions due to decreased electrostatic repulsions between negatively charged phosphodiester linkages. Thus, site-selective DNA scission by ARCUT, which is based on the invasion of pcPNA, is inefficient (as far as molecular crowding conditions are not employed; vide ante). This shortcoming must be solved for further applications, especially in vivo, since the inside of cells is abundant in metal ions (the concentrations of K^+^, Na^+^, and Mg^2+^ in the typical living cells are 140, 10, and 0.5 mM, respectively).

**Figure 5:**
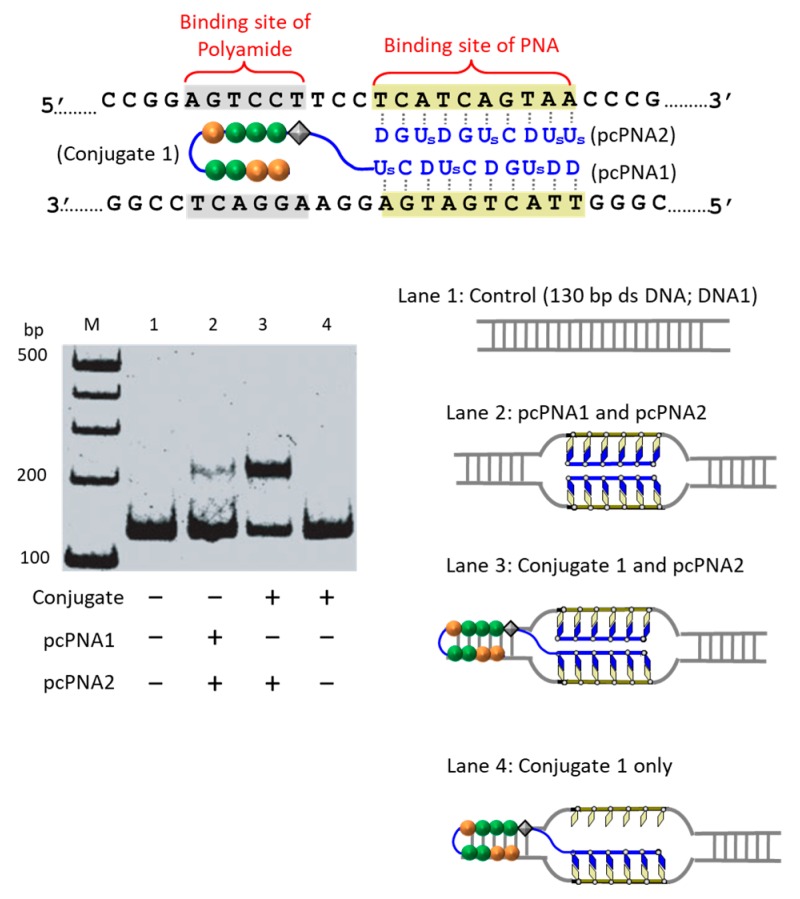
Gel-shift assay for binding of the pcPNA-polyamide conjugate and complementary pcPNA to dsDNA. The pcPNA (blue) binds to 5′-TCATCAGTAA-3′ and the polyamide (orange and green balls) bind to 5′-AGTCCT-3′ in the DNA1. Lane 1, DNA only; lane 2, DNA with two pcPNA strands without the polyamide; lane 3, DNA with the combination of the pcPNA-polyamide conjugate and complementary pcPNA; lane 4, the conjugate only; M, 100 bp ladder. Invasion conditions: [DNA] = 10 nM, [Conjugate 1] = 100 nM, and [pcPNA] = 100 nM at pH 7.0 and 50 °C for 24 h.

The first strategy to solve this problem is to take advantage of the cooperation of double-duplex invasion of pcPNA with minor groove binding of a pyrrole/imidazole (Py-Im) polyamide (Figure 5) [39]. The Py-Im polyamide is known to bind to the minor groove of DNA through multiple hydrogen bonds, and its DNA binding is hardly affected by high salt conditions [40]. The covalent conjugate of pcPNA (blue line) with pyrrole–imidazole hairpin polyamide (orange and green balls) was synthesized and combined with another pcPNA strand (blue line) which is complementary with the pcPNA in the conjugate. Exactly as designed, the binding to double-stranded DNA was satisfactorily strong even under high salt conditions mimicking the inside of living cells ([K^+^] = 140 mM, [Na^+^] = 10 mM, and [Mg^2+^] = 0.5 mM). Upon the addition of Ce(IV)/EDTA, the target site was successfully cut (under the high salt conditions). In this strategy, a 16 bp sequence in DNA was precisely recognized (10 bp by the double-duplex invasion of the two pcPNAs and 6 bp by the minor groove binding of the polyamide.

Conjugation of pcPNA with a nuclear localization signal (NLS) peptide allows the double-duplex invasion to occur under high salt conditions (a PKKKRKV peptide is attached to the terminus of PNA) [41]. Primarily due to electrostatic interactions of the positive charges of NLS with the negative charges of DNA, the invasion activity is enhanced both thermodynamically and kinetically. Upon the treatment with Ce(IV)/EDTA, the target site is selectively hydrolyzed under the conditions where unmodified pcPNAs hardly work. pcPNA-NLS conjugates can be spontaneously incorporated to various cells even without transfection agents, but the transfection is further promoted when they are combined with both a complementary oligonucleotide carrier and a lipofection agent [42]. The conjugates were efficiently localized in nuclei as expected. These features should be advantageous and promising for various in vitro and in vivo applications.

## 6. Site-Selective DNA Cutter Using Only One Strand of PNA

In the first-generation ARCUT, the scission site is activated by double-duplex invasion of two pcPNA strands to DNA. Two pcPNA strands are required to bind to the two DNA strands through Watson-Crick pairing and sufficiently compensate the energy loss due to the partial raveling of double-stranded DNA. In new strategies, however, only one PNA can successfully bind to double-stranded DNA through covalent or non-covalent approaches. By combining these strategies with Ce(IV)/EDTA (or S1 nuclease), a site-selective DNA cutter using only one PNA derivative has been developed (see Section 6.1).

In one-PNA systems, pseudo-complementary bases to suppress the formation of PNA/PNA duplexes are unnecessary. Furthermore, they are advantageous from both kinetic and thermodynamic viewpoints, especially for in vivo applications.

### 6.1. Site-Selective Activation by One Molecule of PNA-NLS Conjugate

The conjugate of PNA with NLS, presented in Section 5.2, also shows unimolecular invasion (not double-duplex invasion) [43]. The invasion site was successfully activated by the invasion and preferentially hydrolyzed by Ce(IV)/EDTA. Conventional PNA without pseudo-complementary bases can be utilized for the conjugate, since there is no competitive homo-hybridization between two additive strands. Thus, the combinations of two pcPNAs in the first-generation ARCUT can be replaced by one strand of PNA-NLS.

The PNA-NLS conjugates are easily transfected into cells without any transfection agent and preferentially localized in nuclei there. Thus, these conjugates are highly promising as antigene agents [44]. Recently, microRNA was satisfactorily targeted by PNA for cancer therapy [45]. Simultaneous administration of two PNAs targeting the corresponding two microRNAs greatly enhanced the pro-apoptotic effects for much more efficient therapy.

### 6.2. Other Strategies for Unimolecular Invasion of PNA

**Figure 6:**
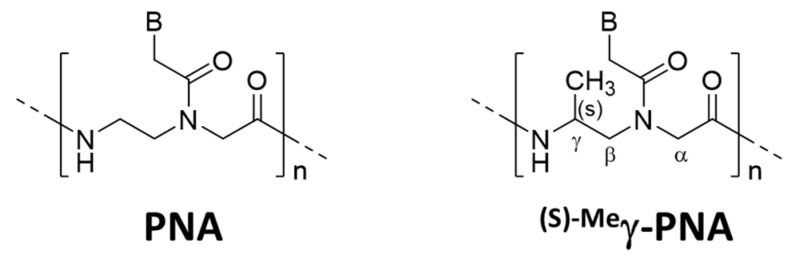
Structure of ^(S)−Me^γ-PNA which shows unique invasion properties. For the purpose of comparison, the parent PNA is also shown.

Several strategies were reported to promote unimolecular invasion of PNA. When an (S)-methyl stereogenic center is introduced to the γ-position of PNA backbone (Figure 6), only one strand can satisfactorily invade to double-stranded DNA [46]. For example, ^(s)−Me^γ-PNA of 15 nucleobases successfully invaded to the complementary site in either strand of double-stranded DNA. There was no specific limitation in the sequence, and the mismatch recognition was satisfactory. This efficient one-strand invasion is ascribed to preorganization of this DNA analog in a right-handed helix, which stabilizes the duplex with complementary DNA. The energy, required to invade to double-stranded DNA, can be satisfactorily paid off by the formation of only one DNA/^(s)−Me^γ-PNA duplex (15 bp). Consistently, the one-strand invasion never occurred when the ^(s)−Me^γ-PNA was shorter (e.g., 10 nucleobases).

Addition of single-stranded DNA binding protein is effective for unimolecular invasion of PNA to double-stranded DNA [47]. There, the protein traps the single-stranded portion, which is formed upon the invasion complex formation. RNase A is also effective to promote unimolecular invasion [48]. The site-specificity of RNase A-promoted unimolecular invasion is satisfactorily high. In contrast, subtilisin A (a protease) is virtually ineffective, although its pI (9.4) is similar to that of RNase A (9.6). These results confirm that the strong binding of the single-stranded DNA portion by the active site of RNase A is crucial for the promotion of the unimolecular invasion.

## 7. Fundamental Information on Double-Duplex Invasion

### 7.1. Promotion of Invasion by the Molecular Crowding Effect

The inside of living cells is largely occupied by proteins and other biomolecules and, thus, its physicochemical properties are much different from the bulk aqueous phase. These intracellular environments (molecular crowding effects) have been successfully simulated by the addition of polyethylene glycol to aqueous solutions. It was shown that molecular crowding conditions greatly promote the invasion of a pair of pcPNA to double-stranded DNA [49]. Under molecular crowding conditions, double-duplex invasion complexes are spontaneously formed at 37 °C. It is noteworthy that the invasion is successful even in high salt media where the invasion otherwise never occurs. Strong potential of the pcPNA invasion for various intracellular applications is indicated. Crucial effects of molecular crowding conditions on the double-duplex invasion have been furthermore evidenced.

According to Sugimoto et al. [50,51], both DNA/DNA duplex and PNA/DNA duplex are destabilized by molecular crowding effects. However, the magnitude of destabilization of DNA/DNA duplex is far larger than the value for the destabilization of PNA/DNA duplex. As the result, double-duplex invasion is promoted by the molecular crowding effect. The destabilization of the DNA/DNA duplex under molecular crowding conditions should be also favorable for spontaneous formation of the invasion complex.

### 7.2. Visual Reconfirmation of PNA Invasion

**Figure 7:**
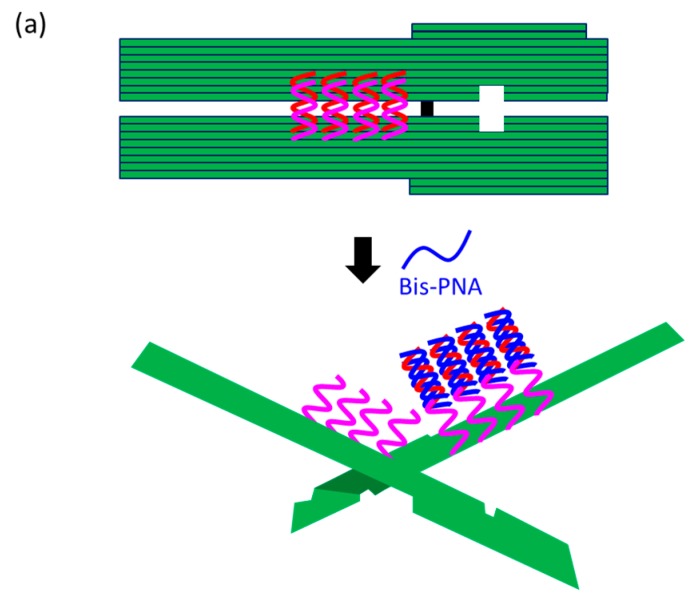

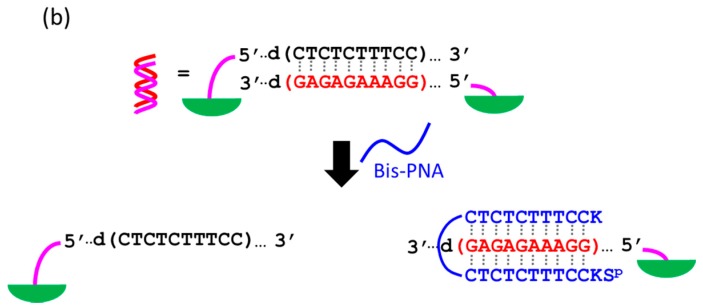
Structures of nanomechanical DNA origami devices used to visually detect PNA invasion. (**a**) Schematic illustration of the system; and (**b**) structures of the pre-closing zipper elements and PNA.

At present, PNA invasion have been detected and analyzed mostly by electrophoretic mobility shift assay. The band shift observed in these assays is mainly due to the induction of a kink or a bend by the invasion. In some cases, however, it is difficult to tell whether the invasion does not really occur or the structural change is simply too small. More universal assay systems are required to facilitate the study of PNA invasion. In order to obtain a more definite conclusion through visual information, a “single-molecule” visual probe was developed using nanomechanical DNA origami pliers (Figure 7) [52]. The device consists of ca. 170 nm lever domains, each of which is made of six antiparallel DNA scaffold helices from M13 (ca. 20 nm wide) [53]. These levers are joined together at a fulcrum via two phosphodiester linkages in the M13 scaffold so that an immobile Holliday junction is formed between the levers. With the use of this device, invasion of a PNA (n-PKCCTTTCTCTC-CTCTCTTTCCK-c) to a consecutive homopurine/homopyrimidine sequence in double-stranded DNA, bound to the levers, was visually analyzed. In the absence of the PNA, the levers are closed due to duplex formation of complementary DNA strands (the top in Figure 7a). When the PNA forms a triplex with the homopurine sequence of the DNA, however, the zipper elements dissociate and “unzip” the pliers (the bottom in Figure 7a). This process was clearly detected by atomic force microscopy in terms of the clear-cut shape transition of DNA pliers from the parallel closed form into the X-shaped open form.

## 8. Conclusions

Site-selective DNA cutters (ARCUT), previously developed by our group, are composed of two strands of pcPNA and Ce(IV)/EDTA complex. For some purposes, S1 nuclease can be used, in place of Ce(IV)/EDTA. Recently, these man-made cutters have been employed for various practical applications. One of the promising examples is to use the genomic fragments for the construction of nanoarchitectures. Since the epigenetic information is kept intact, they should be much different from the ones involving unmodified DNA in structures and other physicochemical properties. At the same time, various covalent and non-covalent modifications of ARCUT have been attempted to promote the invasion and improve the site-selective DNA scission. Some problems of the first-generation ARCUT (e.g., difficulty to cut the G-rich sequence and DNA scission under high salt conditions) have been solved. Site-selective activation of double-stranded DNA by a single PNA strand has been also accomplished to provide new types of site-selective DNA cutters. Furthermore, the “molecular crowding effect”, which characterizes the specific environments in cells, has shown to facilitate the double-duplex invasion. All these results confirm that ARCUT, based on double-duplex invasion of pcPNA, should be highly promising for future applications in biochemistry, biology, medicine, and biotechnology. Compared with currently available protein-based DNA cutters (ZFN, TALEN, and CRISPR-Cas9), ARCUT is completely chemistry-based and advantageous in that any functional group can be straightforwardly introduced through chemical modification. Based on sophisticated molecular design, unprecedented functions, which cannot be exhibited by protein-based cutters, should be accomplished both in vivo and in vitro.
